# Influence of Probiotic Feed Supplement on *Nosema* spp. Infection Level and the Gut Microbiota of Adult Honeybees (*Apis mellifera* L.)

**DOI:** 10.3390/microorganisms11030610

**Published:** 2023-02-28

**Authors:** Ivana Tlak Gajger, Srebrenka Nejedli, Luka Cvetnić

**Affiliations:** 1 Department for Biology and Pathology of Fish and Bees, Faculty of Veterinary Medicine, University of Zagreb, 10 000 Zagreb, Croatia; 2 Department for Anatomy, Histology and Embryology, Faculty of Veterinary Medicine, University of Zagreb, 10 000 Zagreb, Croatia; 3 Department for Bacteriology and Parasitology, Croatian Veterinary Institute, 10 000 Zagreb, Croatia

**Keywords:** honeybee colony, *Apis mellifera* L., bacteria, probiotics, EM^®^ for bees, gut microbiota, *Nosema* spp., colony strength

## Abstract

Honeybees’ gut microbiota can provide new valuable access into the pathogenesis-related factors included in infections. Hence, we researched the presence and comparison of gut microbiota groups in control and *Nosema* spp.-infected honeybee colonies through high-throughput sequencing of the 16S rRNA. As the newest approach in apiary management, we hypothesize that the EM^®^ probiotic for bees could have an important role in therapeutic and immunomodulatory effects on honeybee colonies. The aim of this study was to estimate its impact on the gut microbiota composition of adult honeybees. The major genera were detected, where Lactobacillus was the most abundant genus, followed by Gilliamela, Snodgrassella, and Bifidobacterium. Inoculation with *Nosema* spp. spores made the relative proportions of Bifidobacterium lower, which was ameliorated by EM^®^ for bees’ application. In addition, EM^®^ for bee applied treatments suppressed the increase in the number of *Nosema* spp. spores. This result points out that continuous EM^®^ for bees treatment shall change bees’ gut microbiome composition and mitigate the influence of *Nosema* spp. infection. *Snodgrassella alvi* was a major member of the honeybee gut microbiota and may be significantly increased by long-term treatment with EM^®^ for bees. Toward these results, it is possible that EM^®^ for bees treatment will protect honeybees from herbicide glyphosate negative effects in agricultural fields by improving microbiome and immune functions.

## 1. Introduction

Honeybee colonies (*Apis mellifera* L.) represent the most important social insects. They have a crucial role in plant pollination [1] and are closely linked to global food production and natural biodiversity balance maintaining. Therefore, their economic, ecological, and social values are enormous [2,3]. Due to their specific nutrition physiology and flight behavior patterns, they show complex interactions with environmental ecosystems and consequently with a diverse range of microorganisms [4]. In recent years, large-scale colony declines have been reported. These losses have been associated with unfavorable pedoclimatic and forage frames, parasites and pathogen infections, pesticide intoxications, and nutritional stress of different origins [5,6,7,8,9]. All these factors are often combined by the beekeeper’s management practices [10,11,12]. Consequently, they appear in a meaningful and synergistic package approach, possibly causing different disturbances of the adult honeybee gut microbiota composition.

The core gut microbiota of adult honeybees consists of complex bacteria communities with high genomic diversity whose functionality is dictated by the host, environment, and social exchanges as well as microbial interactions among themselves [4,13,14]. This microbiota is located in various parts of the gastrointestinal tract, including the crop (just a few microorganisms originated from the environment, e.g., *Apilacobacilus kunkei*), midgut (mostly no stable microbiota), ileum (diverse microbial clusters; 10^8^ bacterial cells, e.g., *Snodgrasella alvi*, *Gillimella apicola*, *Lactobacillus* Firm-4 and Firm-5, *Bifidobacterium* spp., *Frischella perrara*, *Bartonella apis*, *Parasaccharibacter apium*, *Bombella apis*, *Apibacter adventoris*, *Apibacter mensalis*), and rectum (10^9^ bacterial cells, e.g., *Lactobacillus* Firm-4, *Lactobacillus* Firm-5, *Bifidobacterium* spp.) [14,15,16,17,18]. Bacterial symbionts or “good bacteria” in the honeybee gut have important functions for host nutrition, food digestion, metabolism, development, weight gain, reproduction, behavioral physiology, and immunity through pathogen and insecticide resistance [19,20,21,22,23,24,25]. It is known that gut microorganisms significantly contribute to pollen digestion, which is the main source of proteins in honeybee nutrition. Therefore, the microbiome composition affects survival rates [26]. In addition, gut microflora have a crucial role in vitamin, fatty acid, and amino acid synthesis [27], in the development and maturation or renewal of enterocytes [28], in the increasing antimicrobial peptide gene expression, pheromone production [29], and in the formation of the biofilm on gut epithelium as a mechanical barrier against disease-causative agents [15].

Dysbiosis in honeybees is often defined as gut–intestinal microbial imbalance linked to a host deficiency, such as deficient development, lower body mass, earlier worker mortality, and general health, metabolism, and fitness status [30]. In addition, in such a situation, the different environmental influences (e.g., the high infection rate of *Nosema ceranae* spores or immune response suppression caused by oxidative stress) could change the composition of gut bacterial phylotypes and remaining microbiome components (e.g., fungi) leading to the appearance of visible clinical signs of opportunistic diseases and colonies weakening [31,32]. Infections of honeybees with the endoparasite *N. ceranae* and with simultaneous exposure to pesticides can significantly contribute to gut dysbiosis [33,34]. In addition, previously published results showed that *N. ceranae* was included in the removal of *Serratia* spp. from bees’ guts, and a consequently significant disturbance in *Snodgrassella* spp. and *Bartonella* spp. was determined [35]. Furthermore, glyphosate combined with *N. ceranae* spore infection change the gut microbiome composition by decreasing the average proportions of *Snodgrassella alvi* and *Lactobacillus apis* [36]. It is interesting that after in vivo treatment of honeybee colonies with oxalic acid, it reduces amplicon sequence variant richness and alters the gut microbiome composition, especially in the genus *Bombella* and bacteria *Lactobacillus kunkeei* [37].

Published studies on the impacts of used commercial probiotics and prebiotics on different aspects of honeybee health are incomplete and contradictory. Some studies show that probiotics increase adult bees’ mortality and different pathogen loads, whereas others suggest that the application of probiotics has a positive effect on the protection against diseases, apian product gains, and the strength of colonies or wax gland development [38,39,40,41,42,43,44,45].

*N. ceranae* is a microsporidium that causes a parasitic disease of honeybee colonies called nosemosis type C and adversely affects adult honeybees’ health by parasitizing in the midgut epithelium and impairing digestion and absorption of nutrients [46]. For efficient beekeeping management and sustainable alternative strategies for nosemosis type C, control is needed, especially today, because there is not a single registered authorized veterinary medicine product for nosemosis control. To facilitate full therapeutic success, there is a need for the appliance of bio-inspired honeybee colony protection products in form of feed supplements [47,48,49,50,51,52] and novel technology designs based on natural ingredients active against microsporidia pathogens. The other possibility is to trigger honeybees’ immune defense responses [53] and, at the same time, avoid the possibility of detection of hazards residual in honey and wax originating from treated hives.

One of the natural and bio-inspired ways to protect honeybees’ health and to produce safe apian products is the use of a mix of effective microorganism cultures and EM^®^ PROBIOTIC FOR BEES (hereinafter, EM^®^ for bees). This commercially available product is a proprietary probiotic formulation owned and managed by the EM Research Organization in Okinawa, Japan. It contains multiple species of lactic acid bacteria, yeast, and photosynthetic bacteria. After promising results for honeybee nosemosis combating in apiary and laboratory-controlled conditions [53], we hypothesized that EM^®^ for bees could have an important effect on gut microbiome content. The aim of this study is to evaluate the therapeutic effects of EM^®^ for bees on *Nosema* spp. infection levels in relation to honeybee colony strength in apiary conditions. In addition, the gut microbiota composition was obtained using next generation sequencing (NGS) analyses to check the differences between experimental and control honeybee colony groups.

## 2. Materials and Methods

### 2.1. Apiary Conditions

#### 2.1.1. Field Test Design, Feed Treatments, and Bee Sampling

The field experiment was conducted for 42 consecutive days (beginning on the 7th of July 2020) at the beeyard situated in the continental part of Croatia (45°56′54.71″ N, 16°37′46.06″ E) after the major honey harvesting season. For performing the test in field conditions, approximately 12 homogeneous honeybee colonies (*A. mellifera carnica,* Pollmann, 1879) were placed in standard Langstroth–Root (LR) hives. Colonies were acquired from the same apiary. Experimental groups were composed of colonies: TH1, colonies were naturally infected with *Nosema* spp. spores; TH2, colonies were treated with EM^®^ for bees; TH3, colonies were additionally infected with *Nosema* spp. spores before the start of treatment with EM^®^ for bees; and TH4, control, noninfected colonies without treatment. Detailed descriptions of the feeding regime and samplings are given in Supplementary Materials.

Experimental honeybee colonies (TH2 and TH3) were additionally fed with a total of 300 mL of sugar syrup prepared in 1:1 proportion of water (L) and sugar (kg) (Virosecer, Croatia) supplemented with a 5% EM^®^ for bees solution consecutively for 10 days from the initial sampling (start of experiment; sampling conducted prior to the first application session). Sugar syrup with probiotic as well as pure sugar syrup (TH1) were applied to honeybee colonies by spraying directly on frames (15 mL of diluted supplemented solution per frame) covered with adult honeybees. The pertaining control honeybee colony (TH4) received only 300 mL of pure sugar syrup prepared and administrated in the described manner. The dose of EM^®^ for bees was adapted according to the manufacturer’s instructions. Throughout the honeybee colonies’ clinical inspection, approximately 60 forager adult bees per colony were sampled from the entrance of the hive for microscope quality and quantity analyses on *Nosema* spp. spores. Adult honeybee samples were collected in clean plastic receptacles by catching bees directly in front of hive entrances using long tweezers. Each sample was taken on the 7th of July, 17th of July, 27th of July, and 20th of August 2020.

At the initial clinical inspection, none of the honeybee colonies showed clinical signs of diseases. In addition, the previously performed treatment against the mites *Varroa destructor* was carried out on the 25th of June 2020 (Apitraz^®^, a.m. amitraz) to avoid the deleterious effects of mite parasitation on honeybee colony health. To the best of our knowledge, no pesticides were used in the surrounding agricultural area during the field experiment.

#### 2.1.2. Sampling of Adult Honeybees’ Guts 

The full length of the intestine of each individual adult honeybee (n = 10 specimens per pooled sample) was extracted after exposure to a low temperature (10 min at 4 °C). For extraction, a larger pair of forceps was used to fix the head and the thorax of each specimen and additionally a smaller pair of forceps to support the top of the last segment of the abdomen where the intestines were carefully pulled out. After the described step, the esophagus, honey sac, and midgut were removed by cutting them off. For examination of the gut microbiome content, gut samples (ileum and rectum) were fixed in the Eppendorf tubes and were cooled directly in a box full of ice during transportation. In the laboratory, gut samples were stored at −80 °C until molecular analyses were performed.

#### 2.1.3. Clinical Inspections of Honeybee Colonies and Strength Estimation

The presence of the honeybee queen, the mortality of the adult bees, as well as clinical signs of brood diseases were inspected during each opening of the hives at the experimental beeyard. The Liebefeld method for visual estimation of the number of adult bees and brood was performed to determine the strength of honeybee colonies [54]. The assessment of honeybee colony strength was conducted on the 1st (7th of July) and the 42nd (20th of August) day of the experiment. The estimation was conducted during the morning hours (around 10:00 a.m.), before massive forage flights of workers.

### 2.2. Laboratory Examinations

#### 2.2.1. Presence Determination and Quantification of *Nosema* spp. Spore Levels

Honeybees were counted in each sample. Their abdomens were separated, thoroughly crushed, and homogenized in a plastic container loaded with 1 mL of pure water per bee specimen. *Nosema* spp. spores were counted in each sample using a Malassez hemocytometer. Infection levels were calculated according to the World Organization for Animal Health (WOAH) guidelines [55]. Each counting procedure was repeated three times. The counting equipment was carefully cleaned after each sample counting to avoid contamination with spores from the previous sample.

#### 2.2.2. Gut microbiota Processing and Analysis

##### Extraction

All honeybee gut samples were processed by homogenization for 1 min in sterile plastic tubes with the addition of 0.5 mL 1 × TE (10 mM Tris-HCl, 1 mM EDTA, pH 8). Total DNA was extracted using the DNeasy PowerSoil Kit (12888-50, Qiagen, Hilden, Germany) according to the manufacturer’s instructions. The DNA extractions were monitored with electrophoresis on a 1% agarose gel to check the purity and then were quantified using a NanoDrop 1000 spectrophotometer (ThermoScientificTM, Waltham, MA, USA). The final concentration of the DNA sample was adjusted to 20 ng/µL.

##### Amplicon Sequencing by NGS

The V3-V4 regions of bacterial and archaeal 16S rRNA were amplified using the Pro341F (CCTACGGGNBGCASCAG)/Pro805R (GACTACNVGGGTATCTAATCC) primers and the dual-index method [56,57]. Amplicons were paired-end sequenced on a 2 × 284 bp cycle using the Illumina MiSeq system with MiSeq Reagent Kit ver 3 (600 Cycle) chemistry and barcoded. Paired-end sequencing readings were connected using a fast-q-join program with default lining [58]. Only linked readings that had a quality value shot of ≥20 for more than 99% of the sequence were extracted using the FASTX-Toolkit [59]. The chimeric sequences were deleted with usearch 6.1 [60,61,62]. Nonchimeric readings were submitted for 16S rDNA-based taxonomic analysis using the Ribosomal Database Project (RDP) Classifier ver. 2.11 (attributing taxon—Phylum 0.8*1) and the TechnoSuruga Lab Microbial Identification database DB-BA ver 13.0 (TechnoSuruga Laboratory, Ltd., Shizuoka, Japan) with homology for ≥97% [63,64].

##### Sequence Analyses

Secondary analyses were conducted based on the results of the core set of the previously mentioned database. For comparative analysis between samples, the software Megagenome@KIN ver 5.0 (World Fusion, Tokyo, Japan) was used. With the purpose of analyzing sequence similarities among different operational taxonomic units (OTUs), multiple sequence alignment was performed by using the free available metagenome analysis software (MEGAN) ver. 2.5.0 and R package multi-comp software ver 4.0.5 environment for statistical computing and graphics [65]. For group comparison analyses, linear discriminant analysis effect size (LEfSe) software ver. 1.0 with the pertaining website was used: https://huttenhower.sph.harvard.edu/galaxy/ (accessed on 16 June 2022).

### 2.3. Statistical Analyses of Colonies’ Strength and Nosema spp. Infection Levels

Data were analyzed with the Stata 13.1. computer program (Stata Corp, College Station, TX, USA). The number of *Nosema* spp. spores detected on a certain date were compared between four groups using the Kruskall–Wallis nonparametric test. Paired comparisons between groups on the same date were conducted by the Dunn test, and the results are expressed as z and *p* values. The number of spores was log normalized and checked for normality of distribution using the Shapiro–Wilk test. Log-transformed data were longitudinally compared between different time points of sampling within the same group using a paired t-test. All results are presented as the main values and their standard deviations. Statistical significance testing was carried out with a significance level of α = 0.05 to define statistical differences (0.95 confidence interval).

## 3. Results

### 3.1. Apiary Conditions

#### Estimated Strength of Honeybee Colonies

**Figure 1:**
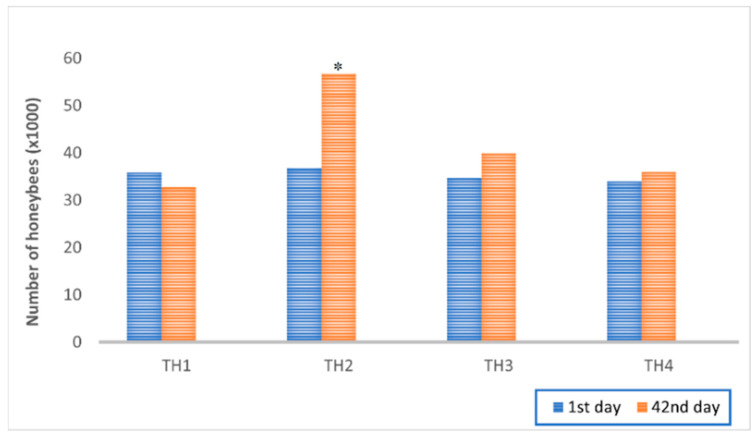
Honeybee colonies’ strength variations between control (TH1, TH4) and experimental groups (TH2, TH3) by the estimation days (1st and 42nd day from the initial day of the experiment); statistically significant difference for TH2 on the second day of estimation vs. TH1, TH3, and TH4; * *p* < 0.05.

Differences in the average number of honeybees *per* group during two estimation dates are shown in Figure 1. Statistically significant differences in honeybee colony strength between the control and experimental groups were determined on day 42 (*p* < 0.001; F = 19.71). A higher number of honeybees was estimated in groups treated with EM^®^ for bees (TH2, TH3) compared to control groups (TH1, TH4). The colony strength of the TH1 and TH4 groups was similar.

### 3.2. Laboratory Examinations

#### 3.2.1. Determination of *Nosema* spp. Infection Levels in Adult Bee Samples

Results of the estimation of *Nosema* spp. infection levels are presented in Table 1. A decline in the number of *Nosema* spp. spores in adult bee samples collected in the colonies fed with EM^®^ for bees (TH2; TH3) in the second (*p* < 0.01; *p* < 0.001) and third (*p* < 0.01; *p* < 0.001) sampling term through a statistically lower number of spores in comparison to the initial sampling before experimental feeding was observed. Only after the last subsequent sampling day (day 44), the increase in the number of spores was estimated for both experimental groups of honeybee colonies. Opposite to the presented results, in the control groups TH1 and TH4, the continuous increase in *Nosema* spp. infection levels was confirmed for each subsequent sampling term, respectively.

Furthermore, in the experimental group TH2, the decreased number of spores compared to the initial spore count was 25.18% on average on day 10, 96% on average on day 20, and 58.10% on average on day 44. For group TH3, the reduction in spore counts in comparison with the initial sampling term, on average, were as follows: 60.51% on day 10 and 40.81% on day 20. Observed differences between groups (TH1, TH2, TH3, and TH4) are statistically significant in every time point of control (Table 2a–d).

#### 3.2.2. The Estimation of Intestinal Microbiota Composition, Richness, and Diversity

**Figure 2:**
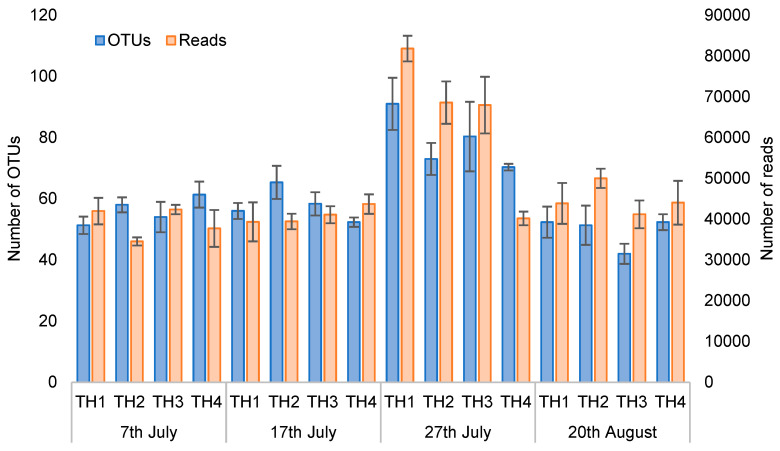
Comparison of the number of OTUs and readings on each treatment and sampling date. Each column in the graph shows the average (n = 3) of the number of OTUs and readings on each treatment and sampling date. Values represent mean ± SEM.

The number of OTUs and readings at the species level for each sampling date and treatment obtained from the amplicon sequence are shown in Figure 2. The number of OTUs and readings on 27th July increased in TH1, TH2, and TH3. However, the number of readings did not increase in TH4. The lifespan of adult summer honeybees is about 42 days, suggesting that most of the July 27th bees were old, and most of the August 20th bees were bees of the next generation.

**Figure 3:**
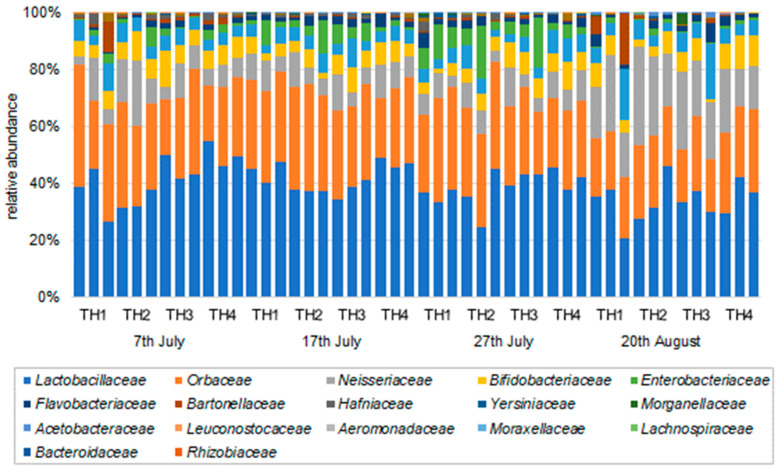
Relative abundance (family) of the gut microbiome in each treatment and on each sampling date.

**Figure 4:**
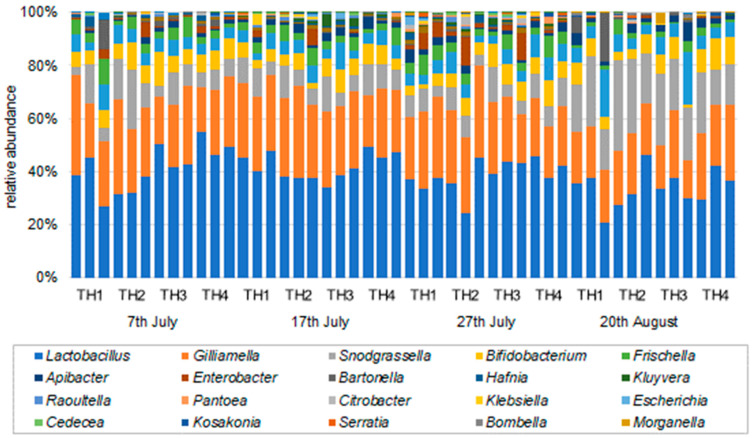
Relative abundance (genus) of the gut microbiome in each treatment on each sampling date.

**Figure 5:**
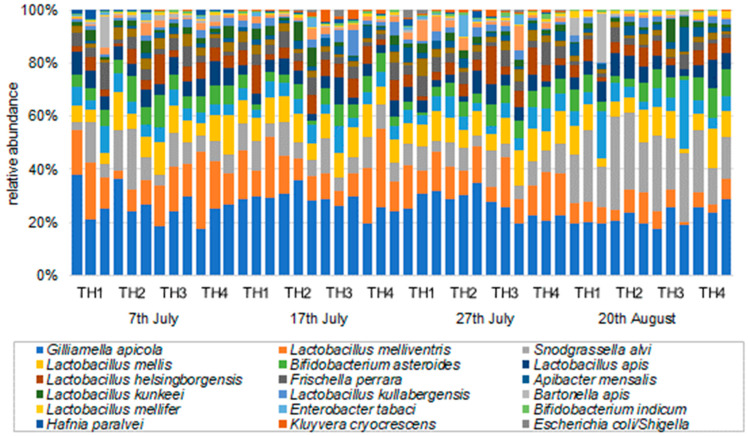
Relative abundance (species) of the gut microbiome in each treatment and on each sampling date.

The bacterial composition ratios (family, genus, and species) determined by the samples of honeybee intestines for each examined sample are shown in Figure 3, Figure 4 and Figure 5. In all groups of honeybee colonies and additional feeding regimes (TH1, TH2, TH3, TH4), the bacteria with the highest compositional ratios were the most common bacteria in the honeybee gut microbiome.

**Figure 6:**
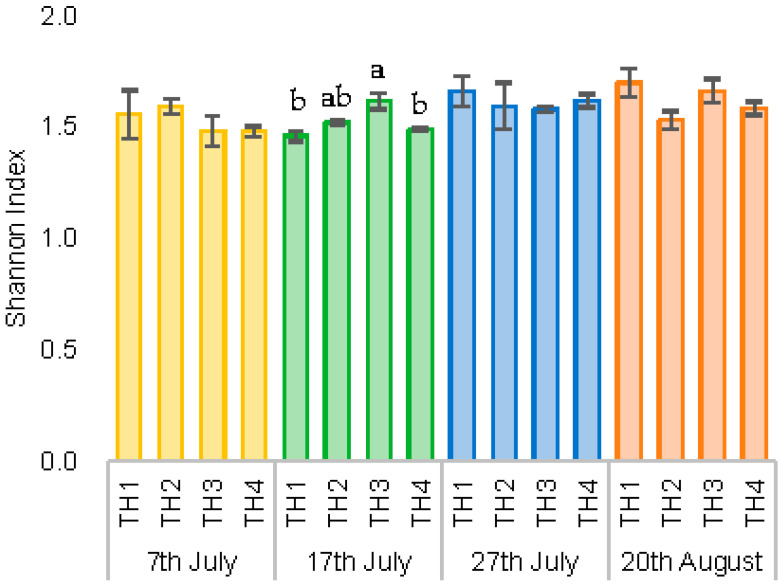
Diversity index (Shannon index) using family data: comparison of treatment intervals and collection dates. There is a significant difference between the days of collection. Values represent mean ± SEM (n = 3). Letters above bars indicate treatments that differ significantly (Tukey HSD test, α = 0.05).

The diversity index (Shanon index) using family-level data for all 48 samples is shown for each sampling date, which was significantly higher for TH3 compared to TH1 and TH4 for the 17th of July data, indicating that TH3 has a higher diversity of gut microbiota. No significant differences were found between treatments on the other sampling dates (Figure 6).

**Figure 7:**
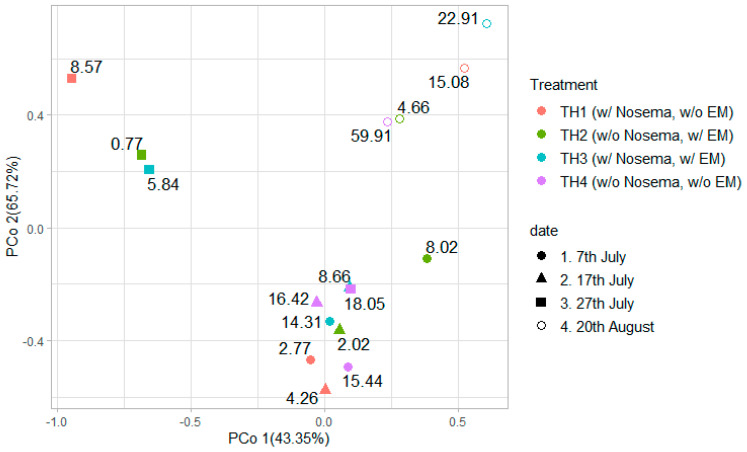
Principal component analysis (PCoA) using the species data comparison of treatment intervals and sample collection dates. Labels in each plot represent the number of *Nosema* spp. spores; w/o—without, w—with.

The results of a principal coordinate analysis (PCoA) visualizing the similarity of complex data (e.g., plotted figures) are shown in Figure 7. The labels of each plot mean the number of *Nosema* spp. spores in the gut of bees. PCoA examined at the species level showed that the plots of the 20th of August samples were away from the other sampling date, further the 27th of July treatments, except for TH4, which was also plotted apart from the other plots. The samples taken on 7th July and 17th July were plotted close together, while TH2 with EM was located slightly further away. In the samples from 27th July, only TH4 was plotted close to the aforementioned samples, while TH2 and TH3 (with EM^®^ for bees treatment) were plotted far from them. This cluster is a relatively low spore count group for *Nosema* spp.

Table 3 shows the percentage of readings of major species to the total readings for each treatment and day with *Gilliamella apicola*, *Lactobacillus meliventris*, *Snodgrassella alvi*, *Lactobacillus melis*, and *Bifidobacterium asteroides*, accounting for a high percentage. The relative proportions of major species tend to vary between treatments and dates.

**Figure 8:**
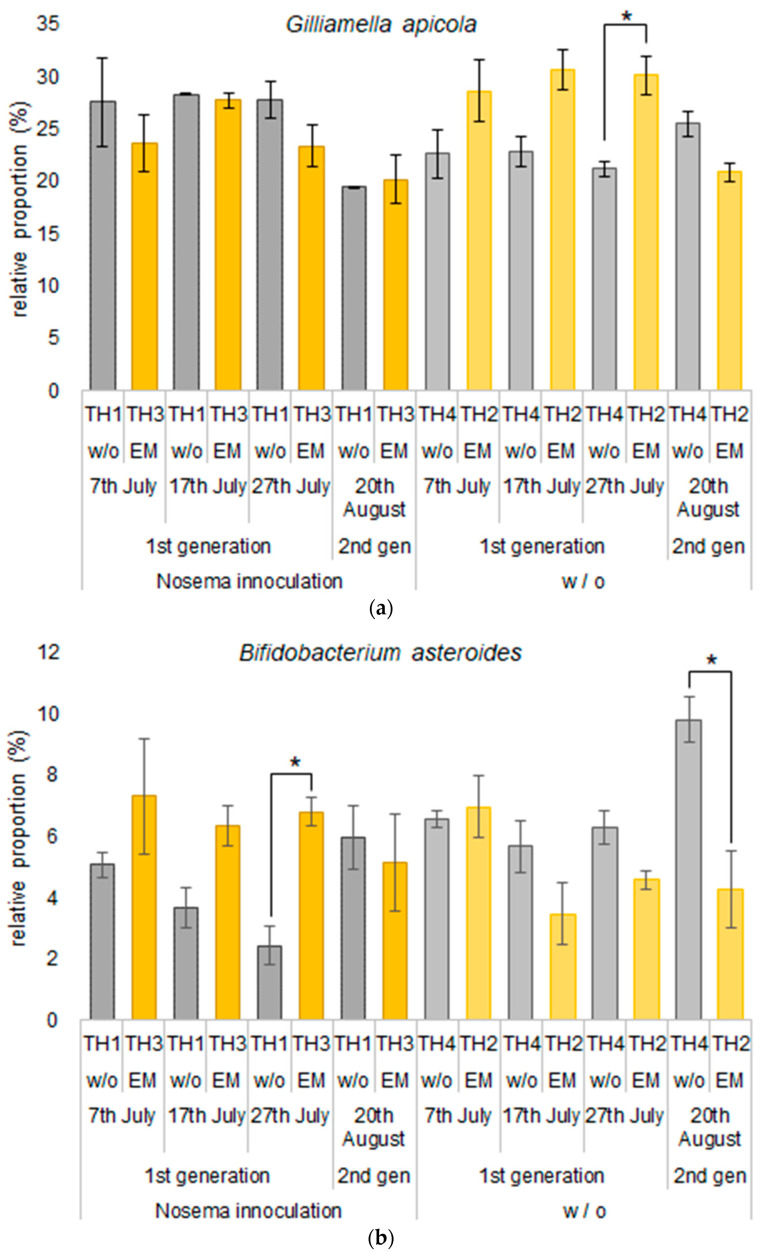
Effect of implemented treatments on the relative proportions of major species: (**a**) *Gilliamella apicola* and (**b**) *Bifidobacterium asteroides*. Values represent mean ± SEM (n = 3). Statistical analysis was conducted using Student’s *t*-test; * *p* < 0.05; w/o—without, w—with.

The effects of *Nosema* spp. spore inoculation and EM^®^ for bees supplement application on the relative proportions of the major species are represented in Figure 8*. Gilliamella apicola*, which accounted for the highest proportion in all treatments, increased the proportion of the intestinal microbiome by inoculation with *Nosema* spores (TH1). On the other hand, of the treatments that were not inoculated with *Nosema* spores, TH4, where spores increased in the gut after infection with *Nosema*, had the lowest proportion of *G. apicola* until 27th July, while TH2 with EM application had the highest relative proportion of *G. apicola* and moreover kept the lowest number of *Nosema* spores in the gut (a). The relative proportion of *Bifidobacterium asteroides* in TH1 inoculated with *Nosema* spp. spores was lower than in TH4 without *Nosema* inoculation, and the proportion continued to decline until 27th July. Even with inoculation with *Nosema* spores, the relative proportion of *B. asteroides* was not low in TH3 with EM application and remained high until 27th July (b).

**Figure 9:**
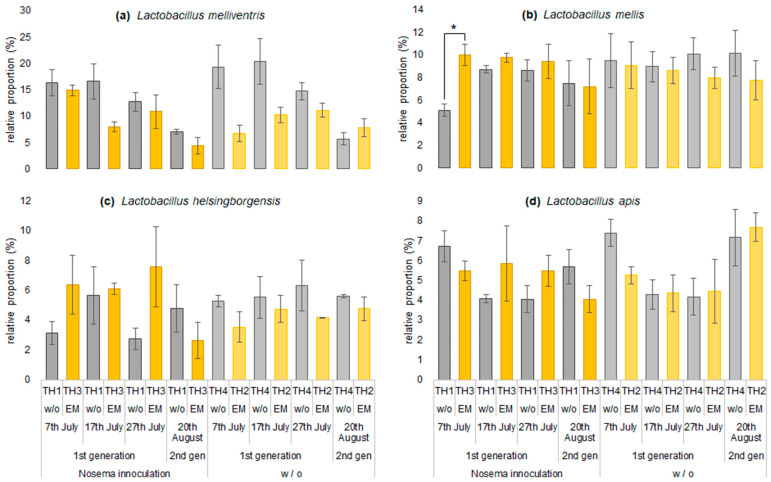
Effect of implemented treatments on the relative proportions of major species: (**a**) *Lactobacillus melliventris*, (**b**) *L. mellis*, (**c**) *L. heisingborgensis*, and (**d**) *L. apis*. Values represent mean ± SEM (n = 3). Statistical analysis was conducted using Student’s *t*-test; * *p* < 0.05; w/o—without, w—with.

The relative proportions of Lactobacillus species in the gut microbiome are shown in Figure 9. *L. melliventris* had a higher relative proportion in group TH4, which had the highest number of *Nosema* spp. spores in the gut in comparison with the other treatments and decreased after 17th July. TH1 decreased similarly, but in group TH2 with only EM^®^ for bees application, it tended to increase, although at a low proportion (a). The relative proportion of *L. mellis* was lower in group TH1 with *Nosema* spp. spore inoculation than in group TH4; however, it was significantly higher in TH3 with in-time EM^®^ for bees application. The proportion changes in *L. mellis* were low (b). The relative proportion of *L. heisingborgensis* was lower in group TH1 than in TH4 but tended to be higher in TH3 fed with EM^®^ for bees. The TH4 proportion increased until 27th July, and TH2 fed with EM^®^ for bees showed the same trend, although at a lower proportion (c). The relative proportion of *L. apis* was high on 7th July and then decreased (TH1 and TH4). In groups TH3 and TH2 treated with EM^®^ for bees, the proportion changes were low until 27th July (d).

**Figure 10:**
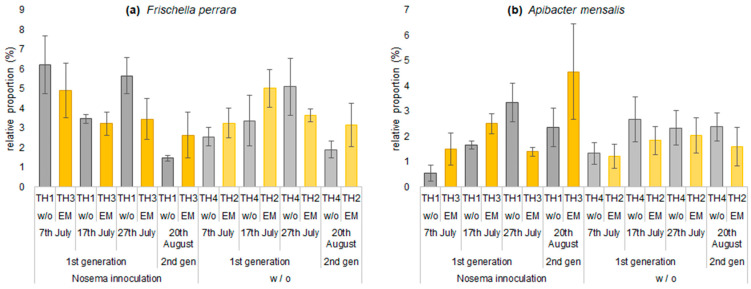
The effect of implemented treatments on the relative proportions of major genera: (**a**) *Frischella perrara* and (**b**) *Apibacter mensalis.* Values represent mean ± SEM (n = 3); w/o—without, w—with.

The relative proportion of *Frischella perrara* tended to be higher in TH1 inoculated with *Nosema* spp. spores than in TH4. The proportion of *F. perrara* increased until 27th July in treatments not inoculated with *Nosema* spp. spores (TH4 and TH2). In the treatments (TH1 and TH3) inoculated with *Nosema* spp. spores, the proportion tended to be lower in TH3 with EM^®^ for bees (Figure 10a). *Apibacter mensalis* in the treatments with inoculation with *Nosema* spp. spores (TH1 and TH3) increased in proportion until 27th July. In contrast, the treatments without inoculation (TH2 and TH4) tended to be stable (Figure 10b).

**Figure 11:**
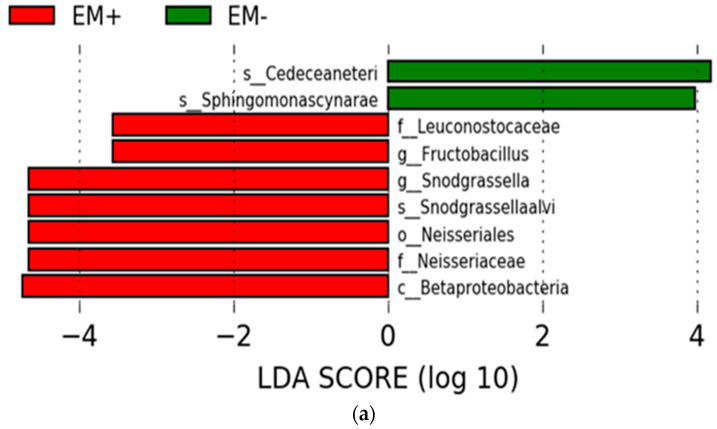

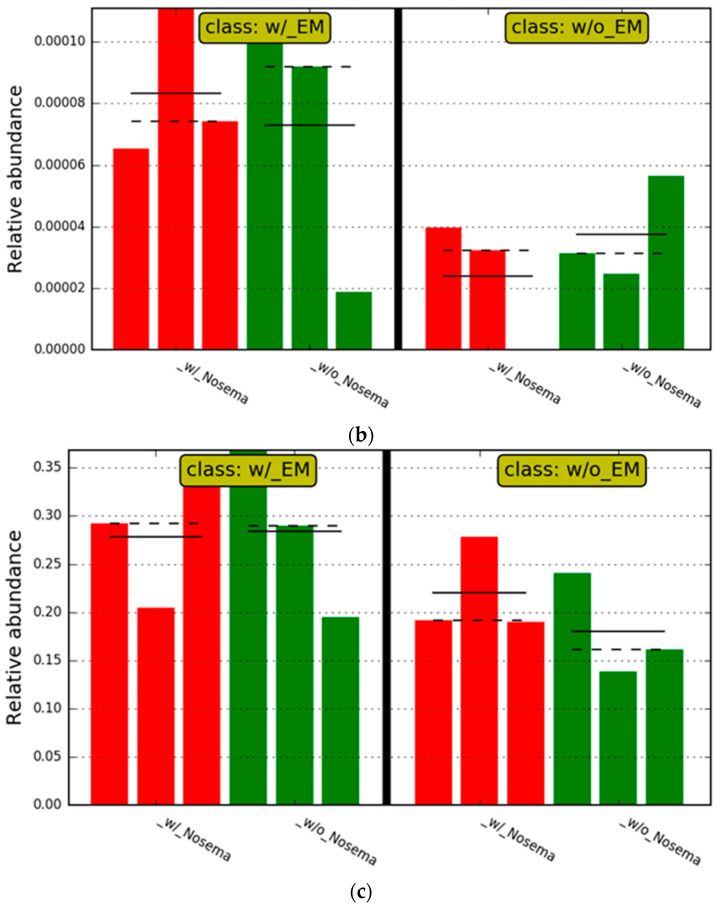
The results of LEfSe (LDA effect size) analysis for samples collected on 20th of August. The histogram of the LDA score shows the biomarkers with statistical differences between the groups (with or without EM^®^ for bees treatment; w/o—without, w—with) (**a**). Histogram of the Fructobacillus (**b**) and *Snodgrassella alvi* (**c**) relative abundance in groups with and without EM^®^ for bee treatments (w/o—without, w—with). Subclasses (with and without *Nosema* spp.) are differentially colored, and the mean and median relative abundance of the biomarkers are indicated with solid and dashed lines, respectively.

The results of LEfSe are shown in Figure 11a,b. LEfSe is an analytical method for extracting microbial groups correlated with differences between groups and for searching for microorganisms responsible for certain phenomena, but after analyzing all samples with and without EM^®^ for bees treatment, it did not show any microorganisms characterizing the differences between the experimental and control groups. When LEfSe analysis was performed just on the samples collected on 20th of August, some microbial groups showed significant differences between the groups with and without EM^®^ for bees treatment (TH2 and TH3, TH1 and TH4) (Figure 11).

The relative abundances of characteristic microorganisms that showed significant differences in the EM^®^ for bees treatment are shown in Figure 11b,c. The relative abundance of Fructobacillus and *Snodgrassella alvi* was significantly increased by supplemental treatment, regardless of the group with and without *Nosema* spp. infection.

## 4. Discussion

The application of EM^®^ for bees has been confirmed by beekeepers as a good management practice in apiaries. In addition, encouraged by the results of the previous study regarding EM^®^ for bees’ impacts on nutritional adult honeybee physiology [53], we considered that it is important to proceed with an investigation on the potential of this complex probiotic to change the gut microbiota composition in adult bees originating from fed colonies.

The strength of colonies was expectedly different between the experimental and control groups of honeybee colonies. In all observation terms, the EM^®^ for bees-supplemented fed colonies were stronger, and the significant difference is most visible on the 42nd day of the experiment. These results are similar to previous scientific records [47,48,66,67,68,69]. The field test was chosen since Nosema-diseased adult bees fed with a natural protein food (pollen, especially beebread) show higher microbiota diversity and stability in comparison with those fed only carbohydrates [70]. In this study, it was again observed that EM^®^ for bees applied as a supplement to sugar syrup decreased microsporidium *Nosema* spp. in the honeybee gut. In detail, in the first three sampling terms, lower spore counts in fed colonies compared with controls were determined, which is in accordance with the results of Tlak Gajger et al. (2020) [53]. Only after the last sampling term on day 44, an increase in the number of spores was estimated for both experimental groups of colonies, probably due to an implemented novel feeding regime. In the pertaining control groups (TH1 and TH4), a continuous increase in *Nosema* spp. infection levels was confirmed for each subsequent sampling term, respectively, which was within the expected ranges under the field, environmental, and study conditions.

According to previously published results, the Lactobacillus and Bifidobacterium microorganisms decrease invasion levels of *Nosema* spp. spores in *A. mellifera* [71] and in *A. cerana* [72]. The fed supplementation with *Parasaccharibacter apium* was confirmed to be a good way to improve the resistance of adult bees to Nosema type C invasion [73]. In addition, some other apiculture positive impacts, such as a decrease in varrosis damage, successful honeybee queen production [74], maintaining good colony vitality status [42,75], and increased honey production [74,76], were reported. In this study, *Nosema* spp. spore counts were higher in accidentally naturally infected groups of colonies TH4 than in colonies that were initially naturally infected with spores of *Nosema* spp., TH1 (Table 1), but the relative proportion of Bifidobacteria was lower in TH1, presumably due to longer *Nosema* spp. invasion. It was inferred that when with *Nosema* spp. spores highly infect adult bees that are young enough, the effect on the gut microbiota is greater, and the relative proportion of Bifidobacterium is reduced. EM^®^ for bees treatment was shown to have the potential to mitigate the effects of nosemosis on the gut microbiota content. However, since high levels of *Nosema* spp. spores were detected also in group TH4, where the relative proportion of Bifidobacterium was kept relatively high, it is assumed that Bifidobacterium does not directly inhibit the formation of *Nosema* spp. spores.

When the samples were compared in time series, those taken on the 20th of August, the second generation of adult honeybees, showed a different microbiome content trend from the earlier taken samples (Figure 6). In addition, when the data of each treatment area for the samples taken on the 20th of August were analyzed, there were significant differences in some microbial groups between the samples from the experimental and control groups (Figure 11). For example, *Snodgrassella alvi*, which has been shown to be a characteristic fungus in the honeybee colonies treated with EM^®^ for bees (TH2 and TH3), was higher in the last sampling term (20th of August) in comparison with other treatments (Figure 11). *S. alvi*, which in this study showed significant differences in the experimental and control honeybee colony groups, is a known major member of the honeybee gut microbiota. This bacterium is susceptible to the herbicide glyphosate, and research has shown that glyphosate consistently reduces this bacterium, resulting in lower resistance to pathogens in honeybees [77] and causing metabolic-level perturbation [78]. In addition, the reduction in this bacterium may be a factor that decreases the immunity of honeybees [77].

The number of outs and readings obtained from amplicon sequencing increased on the 27th of July in groups TH1, TH2, and TH3. However, it did not increase in readings for group TH4 (Figure 2). Therefore, the number of intestinal *Nosema* spp. spores suppressed the increase in the number of gut bacteria more than the fresh natural infection with *Nosema* spp. spores. Then, the group TH2 plot on the 7th of July was slightly off, suggesting that the adult honeybee intestinal microbiota was affected by EM^®^ for bees’ application. The plots of the sampled bees’ gut microbiomes in groups TH1, TH2, TH3, and TH4 on the 20th of August were away from the ones of the other sampling dates. Furthermore, the plots of experimental colonies from groups TH1 and TH3 that were invaded with *Nosema* spp. spores and the ones considered uninvaded (TH2 and TH4) were close together, respectively. Therefore, it is suggested that the gut microbiomes of adult bees sampled on the 20th of August were affected by the invasion with *Nosema* spp. spores more than the level of invasion.

The number of intestinal *Nosema* spp. spores in group TH2 applied only EM^®^ for bees remained lowest, and the relative proportions of Gilliamella were kept higher than in the other groups of colonies. On the other hand, the number of *Nosema* spp. spores remained highest in group TH4, and the relative proportions of Gilliamella were kept lower in the first three sampling terms (Figure 8a). Therefore, EM^®^ for bees’ application to honeybees increased the proportion of Gilliamella in the bees’ gut microbiomes, indicating that it may inhibit the development of *Nosema* spp. Spores in the midgut.

In groups, TH1 and TH3 invaded with *Nosema* spp. Spores, the level of invasion increased until the 27th of July in TH1 and decreased in TH3 (Table 1). *Bifidobacterium asteroides*, *Lactobacillus mellis*, *L. heisingborgensis,* and *L. apis* were higher in group TH3 than in group TH1 until the 27th of July. We think that probiotics may have suppressed the increase in *Nosema* spp. spore levels in the intestine. Both of these proportions in group TH3 decreased on 20th August, and *Nosema* spores increased, probably as a consequence of the long period passed from initial feeding. On the other hand, group TH2 had the lowest number of *Nosema* spp. spores for all performed sampling terms after the 17th of July and the highest relative proportion of *Gilliamella apicola* until the 27th of July and *Snodgrassella alvi* on the 20th of August.

The relative proportions of *Frischlla perrara* and *Apibacter mensalis* on the 27th of July were higher in group TH1 than in group TH3. Furthermore, the number of *Nosema* spp. spores was also higher. However, on the 20th of August, the proportions of these bacteria were higher in group TH3, and the number of spores was also higher in TH3. Thus, if invaded with *Nosema* spp. spores, these bacteria may be associated with the development of *Nosema* spp. spores in the intestine.

*Fructobacillus fructosus*, a member of the genus Fructobacillus, has been reported to exhibit antagonistic activity against the pathogen bacterium *Paenibacillus larvae*, the causal agent of American foulbrood [79]. However, this species is not known from the results of the LEfSe analysis in this study but represents a good topic for further research. It is known that hive structure, environment, and natural diet influence the assembly and maintenance of honeybee gut microbiota and facilitate future experimental designs [18]. According to our knowledge, this is the first study aimed at investigating the impacts of the probiotic EM^®^ for bees on adult bees’ health and immunological conditions by analyzing intestinal microbiome content.

## 5. Conclusions

There have been many reports from beekeepers that honeybee colonies become more resistant to diseases after using EM^®^ for bees. According to the results of this study, the applied probiotic treatments suppressed the influence of *Nosema* spp. infection and changed bees’ gut microbiome composition. In addition, it is probable that the continued use of EM^®^ for bees improves their gut microbiome and, consequently, immunity by maintaining and increasing the level of *S. alvi*. If the higher level of *S. alvi* is supported by feed supplementation with EM^®^ for bees, it may be possible for this beekeeping management practice to reduce the negative effect of glyphosate on honeybees.

## Figures and Tables

**Table 1:** The number of *Nosema* spp. spores per honeybee during supplemental feeding in apiary conditions; mean values ± SD.

Group		Spores of *Nosema* spp. (×10^6^)			
		7th July	17th July	27th July	20th August
TH1 (^1^N = 9)	Mean	2.77	4.26	8.57	15.08
	SD	1.23	2.67	6.19	3.65
TH2 (^1^N = 9)	Mean	8.02	2.02	0.77	4.66
	SD	10.19	2.88	0.42	3.02
TH3 (^1^N = 9)	Mean	14.31	8.66	5.84	22.91
	SD	8.71	5.68	4.71	11.88
TH4 (^1^N = 9)	Mean	15.44	16.42	18.05	59.91
	SD	12.38	10.62	10.53	65.53
*p* (Kruskall–Wallis)		0.001	0.0005	0.0001	0.0001

^1^ Sample from each colony was examined in triplicate.

**Table 2:** Statistics of *Nosema* spp. spore burden differences between the same group for each sampling time point: 7th July (a), 17th July (b), 27th July (c), and 20th August (d).

TH	z	*p*	TH	z	*p*	TH	z	*p*	TH	z	*p*
1:2	−0.581781	0.2804	1:2	1.354283	0.0878	1:2	3.214710	0.0007	1:2	2.350559	0.0094
1:3	−3.020785	0.0013	1:3	−1.544554	0.0612	1:3	0.705668	0.2402	1:3	−1.387949	0.0826
1:4	−3.199795	0.0007	1:4	−2.585449	0.0049	1:4	−1.680162	0.0465	1:4	−2.350559	0.0094
2:3	−2.439005	0.0074	2:3	−2.898836	0.0019	2:3	−2.509042	0.0061	2:3	−3.738509	0.0001
2:4	−2.618014	0.0044	2:4	−3.939731	0.0000	2:4	−4.894872	0.0000	2:4	−4.701119	0.0000
3:4	−0.179010	0.4290	3:4	−1.040895	0.1490	3:4	−2.385830	0.0085	3:4	−0.962610	0.1679
	(a)			(b)			(c)			(d)	

**Table 3:** Relative proportions (%) of the major species in the gut microbiome.

GenBank Identification	7th July				17th July				27th July				20th August			
	TH1	TH2	TH3	TH4	TH1	TH2	TH3	TH4	TH1	TH2	TH3	TH4	TH1	TH2	TH3	TH4
*Gilliamella apicola*	27.6	28.7	23.6	22.7	28.3	30.7	27.7	22.9	27.8	30.1	23.3	21.2	19.4	20.9	20.2	25.5
*Lactobacillus melliventris*	16.4	6.7	14.9	19.3	16.6	10.2	8.0	20.4	12.7	11.1	10.8	14.7	7.0	7.9	4.4	5.7
*Snodgrassella alvi*	7.5	15.6	8.2	6.8	8.5	7.9	7.9	9.4	6.8	7.0	7.7	9.2	20.1	27.0	23.9	17.1
*Lactobacillus mellis*	5.1	9.1	10.0	9.5	8.7	8.6	9.7	8.9	8.6	8.0	9.4	10.1	7.5	7.7	7.2	10.1
*Bifidobacterium asteroides*	5.1	7.0	7.3	6.6	3.7	3.5	6.3	5.7	2.4	4.6	6.8	6.3	6.0	4.3	5.2	9.8
*Lactobacillus apis*	6.7	5.3	5.5	7.4	4.1	4.4	5.9	4.3	4.0	4.4	5.5	4.2	5.7	7.7	4.0	7.2
*Lactobacillus helsingborgensis*	3.1	3.5	6.4	5.3	5.7	4.7	6.1	5.5	2.7	4.1	7.6	6.3	4.8	4.8	2.6	5.6
*Frischella perrara*	6.2	3.2	4.9	2.6	3.5	5.0	3.2	3.4	5.7	3.6	3.5	5.1	1.5	3.2	2.6	1.9
*Lactobacillus kimbladii*	1.4	3.8	3.3	2.5	3.1	2.4	1.7	2.4	3.4	1.9	3.5	2.7	1.7	2.4	1.9	1.9
*Apibacter mensalis*	0.5	1.2	1.5	1.3	1.7	1.8	2.5	2.7	3.3	2.0	1.4	2.3	2.4	1.6	4.6	2.4
*Lactobacillus kunkeei*	2.2	2.7	1.7	2.5	2.4	4.9	0.8	1.6	1.8	2.6	1.4	1.5	1.1	1.4	4.4	1.6
*Lactobacillus kullabergensis*	1.1	1.5	1.9	2.4	2.5	1.5	4.9	2.7	1.9	2.1	2.5	0.8	1.6	1.7	1.0	1.6

## Data Availability

Data are presented in the manuscript.

## Supplementary Materials

The following supporting information can be downloaded at: https://www.mdpi.com/article/10.3390/microorganisms11030610/s1. Table S1 Field test details of feeding regime and sampling.
