# Supplementary material for: Influence of Probiotic Feed Supplement on Nosema spp. Infection Level and the Gut Microbiota of Adult Honeybees (Apis mellifera L.)
Source: Microorganisms. 2023 Feb 28;11(3):610. doi: 10.3390/microorganisms11030610 (PMC10056795; doi:10.3390/microorganisms11030610)
Supplement: Supplementary file 1 [file microorganisms-11-00610-s001.zip › microorganisms-2219848-supplementary.pdf]

Table S1. Field test details of feeding regime and sampling

No.	Sampling date	Treatment*	Colony No.	No.	Sampling date	Treatment*	Colony No.
**1**	7.7	TH1	4A	**25**	7.27	TH1	4A
**2**	7.7	TH1	5A	**26**	7.27	TH1	5A
**3**	7.7	TH1	8A	**27**	7.27	TH1	8A
**4**	7.7	TH2	1A	**28**	7.27	TH2	1A
**5**	7.7	TH2	2A	**29**	7.27	TH2	2A
**6**	7.7	TH2	3A	**30**	7.27	TH2	3A
**7**	7.7	TH3	31	**31**	7.27	TH3	31
**8**	7.7	TH3	34	**32**	7.27	TH3	34
**9**	7.7	TH3	37	**33**	7.27	TH3	37
**10**	7.7	TH4	10A	**34**	7.27	TH4	10A
**11**	7.7	TH4	22	**35**	7.27	TH4	22
**12**	7.7	TH4	29	**36**	7.27	TH4	29
**13**	7.17	TH1	4A	**37**	8.20	TH1	4A
**14**	7.17	TH1	5A	**38**	8.20	TH1	5A
**15**	7.17	TH1	8A	**39**	8.20	TH1	8A
**16**	7.17	TH2	1A	**40**	8.20	TH2	1A
**17**	7.17	TH2	2A	**41**	8.20	TH2	2A
**18**	7.17	TH2	3A	**42**	8.20	TH2	3A
**19**	7.17	TH3	31	**43**	8.20	TH3	31
**20**	7.17	TH3	34	**44**	8.20	TH3	34
**21**	7.17	TH3	37	**45**	8.20	TH3	37
**22**	7.17	TH4	10A	**46**	8.20	TH4	10A
**23**	7.17	TH4	22	**47**	8.20	TH4	22
**24**	7.17	TH4	29	**48**	8.20	TH4	29

* Treatments:

TH1 – honeybee colonies were naturally infected with *Nosema* spp. spores

TH2 – honeybee colonies naturally infected with *Nosema* spp. spores and treated with EM® for bees

TH3 – honeybee colonies were additionally infected with *Nosema* spp. spores before the start of treatment with EM® for bees

TH4 - control, non-infected honeybee colonies without treatment
